# The Effects of Alpha-Linolenic Acid on the Secretory Activity of Astrocytes and *β* Amyloid-Associated Neurodegeneration in Differentiated SH-SY5Y Cells: Alpha-Linolenic Acid Protects the SH-SY5Y cells against *β* Amyloid Toxicity

**DOI:** 10.1155/2020/8908901

**Published:** 2020-08-05

**Authors:** Anna Litwiniuk, Anita Domańska, Magdalena Chmielowska, Lidia Martyńska, Wojciech Bik, Małgorzata Kalisz

**Affiliations:** ^1^Department of Neuroendocrinology, Centre of Postgraduate Medical Education, Marymoncka 99/103, 01-813 Warsaw, Poland; ^2^Department of Physiological Sciences, Institute of Veterinary Medicine, Warsaw University of Life Sciences (SGGW), Nowoursynowska 159, 02-776 Warsaw, Poland

## Abstract

Alzheimer's disease (AD) is the most common neurodegenerative disorder. Amyloid *β*- (A*β*-) induced mitochondrial dysfunction may be a primary process triggering all the cascades of events that lead to AD. Therefore, identification of natural factors and endogenous mechanisms that protect neurons against A*β* toxicity is needed. In the current study, we investigated whether alpha-linolenic acid (ALA), as a natural product, would increase insulin and IGF-I (insulin-like growth factor I) release from astrocytes. Moreover, we explored the protective effect of astrocytes-derived insulin/IGF-I on A*β*-induced neurotoxicity, with special attention paid to their impact on mitochondrial function of differentiated SH-SY5Y cells. The results showed that ALA induced insulin and IGF-I secretion from astrocytes. Our findings demonstrated that astrocyte-derived insulin/insulin-like growth factor I protects differentiated SH-SY5Y cells against A*β*_1-42_-induced cell death. Moreover, pretreatment with conditioned medium (CM) and ALA-preactivated CM (ALA-CM) protected the SH-SY5Y cells against A*β*_1-42_-induced mitochondrial dysfunction by reducing the depolarization of the mitochondrial membrane, increasing mitochondrial biogenesis, restoring the balance between fusion and fission processes, and regulation of mitophagy and autophagy processes. Our study suggested that astrocyte-derived insulin/insulin-like growth factor I suppresses A*β*_1-42_-induced cytotoxicity in the SH-SY5Y cells by protecting against mitochondrial dysfunction. Moreover, the neuroprotective effects of CM were intensified by preactivation with ALA.

## 1. Introduction

Alzheimer's disease (AD) is the most common neurodegenerative disorder being the principal cause of dementia among the elderly. The major pathological hallmarks of AD are senile plaques and neurofibrillary tangles (NFTs) along with the loss of neurons and synapse in the AD brains [1]. Most of these changes appear in the brains of patients long before the onset of clinical symptoms of cognitive decline [2]. A recent study demonstrated that besides these know hallmarks, mitochondrial malfunctions may play a distinct role in AD [3].

Mitochondria are highly dynamic organelles with morphology and numbers being regulated by fission and fusion proteins [4]. The balance between fusion and fission processes is essential to maintain the health of the neuronal cells. Both processes are largely regulated by the guanosine triphosphatase (GTPase) enzymes. Mitochondrial fusion is regulated by the GTPases including mitofusin 1 (Mfn1), mitofusin 2 (Mfn2), and optic atrophy protein 1 (OPA1), and helps to maintain tubular mitochondrial network and optimal mitochondrial functions. Whereas the fission process is regulated by dynamin-related protein-1 (Drp1) and mitochondrial fission 1 protein (Fis1). Excessive mitochondrial fission leads to impaired mitochondrial function and neuronal death in AD [5]. The fusion/fission process is a part of the mitochondrial biogenesis in which the cells increase their mitochondrial mass. However, the mitochondrial biogenesis, another key mitochondrial function, is also impaired in AD. The level of proteins regulating the mitochondrial biogenesis such as peroxisome proliferator-activated receptor gamma coactivator 1-alpha (PGC-1*α*), nuclear respiratory factor 1 and 2 (NRF1, NRF2), and mitochondrial transcription factor A (mTFA) was found to be significantly reduced in human AD hippocampus and cellular models overexpressing APP Swedish mutation [6]. Due to the hypothesis that Amyloid *β*- (A*β*-) induced mitochondrial dysfunction may be the primary process triggering all the cascades of events that lead to AD, identification of the natural factors and the endogenous mechanisms that protect neurons against A*β* toxicity is needed. It has been suggested that neurons may be protected against A*β*-induced damage through activation of the insulin and insulin-like growth factor I (IGF-I) signaling pathways [7, 8]. In recent years, it has been reported that physiological protection against A*β*-induced damage can be mediated by astrocyte-derived insulin and IGF-I [8]. Moreover, the protective role of IGF-I and insulin has been confirmed in animal models of AD [9, 10].

Additionally, growing attention has been paid to the search for compounds from natural sources that can protect neurons against A*β*-induced mitochondrial and synaptic toxicities. Alpha-linolenic acid (ALA) is the plant-derived *ω*-3 fatty acid. Previous studies showed that consumption of dietary ALA reduced the risk of cardiovascular disease and stroke [11, 12]. Gao et al. demonstrated that long-term dietary intake of ALA improved the cognitive function through the activation of extracellular signal-regulated kinases (ERK) and Akt signaling in the aged-rat model [13]. However, the direct effect of ALA on the secretory activity of astrocytes has not been studied yet. Moreover, very little data have been reported on the effects of astrocytes-derived insulin/IGF-I on A*β*-induced mitochondrial dysfunction.

Therefore, in the current study, we investigated whether ALA would increase insulin and IGF-I secretion from astrocytes. Moreover, we explored the protective effect of astrocytes-derived insulin/IGF-I on A*β*-induced neurotoxicity, with special attention paid to their impact on mitochondrial function of differentiated SH-SY5Y cells.

## 2. Materials and Methods

### 2.1. Reagents and Antibodies

Alpha-linolenic acid (ALA), Human Beta Amyloid 1-42 (A*β*_1-42_), All-trans retinoic acid (RA), Insulin, Human Insulin ELISA Kit, Human IGF-I ELISA Kit, 3-(4,5-dimethylthiazol-2-yl)-2-5-diphenyltetrazolium bromide (MTT), bovine serum albumin (BSA), and acridine orange (AO) were purchased from Sigma Aldrich, Saint Louis, MO, USA. Insulin Degrading Enzyme (IDE) was purchased from Abcam Inc., USA. Antibodies to the following targets were used: rabbit anti-TOMM20 antibody (Abcam Inc.), mouse anti-PARKIN (Abcam Inc.), rabbit anti-Synaptophysin (GeneTex, Inc., Alton Pkwy Irvine, CA, USA), mouse anti-*β*3-Tubulin (TUJ 1; Santa Cruz Biotechnology, Dallas, CO, USA), horseradish peroxidase- (HRP-) conjugated goat anti-rabbit IgG antibodies (Abcam Inc.), HRP-conjugated goat anti-mouse IgG antibodies (Abcam Inc.), Alexa Fluor™488-labeled chicken anti-mouse IgG (Thermo Fisher Scientific, Waltham, MA, USA), Alexa Fluor™568-conjugated goat anti-rabbit IgG (Thermo Fisher Scientific).

### 2.2. A*β* Preparation

Amyloid *β*_1-42_ (A*β*_1-42_) was prepared as described previously [14]. Dried synthetic A*β*_1–42_ peptide (Sigma Aldrich) was first dissolved in dimethylsulfoxide (DMSO) and then diluted in phosphate buffer saline (PBS) to obtain a 250 mM stock solution. This solution was incubated at 4°C for at least 24 h and stored at -80°C. Before use, the solution was centrifuged at 12 000 g for 10 min, and the supernatant was used as an oligomeric A*β*_1-42_.

### 2.3. Cell Cultures

#### 2.3.1. Human Astrocyte Cells Cultures

The Normal Human Astrocytes (NHA) were obtained from the Lonza Basel, Switzerland. The cells were cultured in ABM™ Basal Medium (NHA medium, Lonza Basel) supplemented with AGM™ SingleQuots^TM^ Supplements (Lonza Basel) required for growth of Astrocytes.

#### 2.3.2. Preparation of Astrocyte-Conditioned Medium (CM) and ALA-Preactivated CM (ALA-CM)

**Figure 1:**
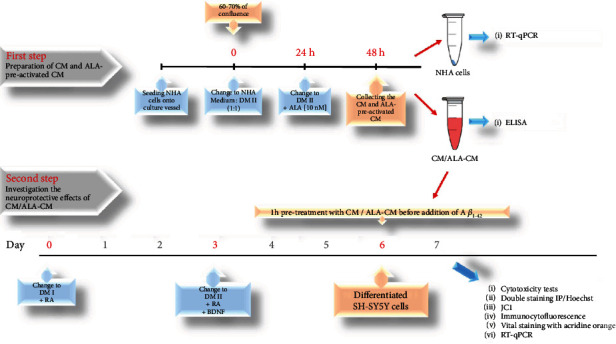
Scheme of the experimental procedures. The first step of the research involved the preparation of astrocyte-conditioned medium (CM) and Alpha-linolenic acid-preactivated CM (ALA-CM). The Normal Human Astrocytes (NHA) cells were allowed to grow until 60-70% confluence. Then, NHA cells were grown in the medium containing NHA-Medium and neurobasal medium (the phase II differentiation medium DM II) (1 : 1). After 24 h, the medium was replaced with DM II medium alone or with 10 nM ALA for the next 24 h incubation to obtain CM and ALA-preactivated-CM. The second step included the evaluation of the neuroprotective effect of CM and ALA-CM on Amyloid *β*_1-42_- (A*β*_1-42_-) induced neurodegeneration of differentiated SH-SY5Y cells. On the day 6th, the SH-SY5Y cells (differentiated) were pretreated for 1 h with CM or ALA-CM before the addition of 5 *μ*M A*β*_1-42_.

After seeding, the NHA cells were allowed to grow for 24–48 hours or until 60-70% confluence in six-well plates. Next, NHA cultures were grown in the medium containing NHA-Medium and neurobasal medium (the phase II differentiation medium DM II) (1 : 1). After 24 h, the medium was replaced with DM II medium. The NHA cells were grown in medium without or with ALA at different doses (10 nM, 50 nM, 100 nM, 250 nM) for 24 h to obtain ALA-preactivated CM (ALA-CM) and control CM, respectively. A dose of 10 nM ALA was used for the following experiments (Figures 1).

#### 2.3.3. Human Neuroblastoma SH-SY5Y Cell Cultures

The human SH-SY5Y cells were purchased from the European Collection of Cell Cultures (ECAAC, cat.94030304, passage 11). The cells were initially cultured in growth media (GM), constituted by Minimum Essential Medium Eagle (MEM, Sigma Aldrich) supplemented with 15% (*v*/*v*) heat-inactivated fetal bovine serum (hiFBS), 2 mM L-glutamine (Sigma Aldrich), 50 IU/mL/50 *μ*g/mL penicillin/streptomycin (Sigma Aldrich), 20 *μ*g/mL gentamicin sulphate (Biowest, Nuaillé, France); and 1 *μ*g/mL Fungizone/amphotericin B (Biowest), at 37°C in a humidified atmosphere containing 5% CO_2_, and kept below ECAAC passage +15 to avoid cell senescence [15].

*(1) Differentiation and Treatments*. The differentiation of the SH-SY5Y cells was carried out in two steps using phase 1 (DM I) and phase 2 (DM II) media. The presented differentiation protocol was modified from Forster et al. [15] and Mackenzie et al. [16]. The DM I medium was MEM (Sigma Aldrich) containing 2.5% (*v*/*v*) hiFBS, 2 mM L-glutamine (Sigma Aldrich), 50 IU/mL/50 *μ*g/mL penicillin/streptomycin (Sigma Aldrich). The DM I was supplemented with 10 *μ*M RA (Sigma Aldrich) immediately before adding the medium to the cells. The DM II medium was Neurobasal medium minus phenol red (Invitrogen, Life Technologies, Saint Aubin, France), supplemented with 2 mM Glutamax I (Invitrogen, Life Technologies), 1x B-27 supplement (50x, Invitrogen, Life Technologies), 20 mM KCl (Sigma Aldrich), and 50 IU/mL/50 *μ*g/mL penicillin/streptomycin (Sigma Aldrich). The DM II was supplemented with 10 *μ*M RA (Sigma Aldrich) and 50 ng/mL Human BDNF (PeproTech EC, Ltd, London, UK) immediately before adding the medium to the cells. On the day 6th, the SH-SY5Y cells (differentiated) were pretreated for 1 h with CM or ALA-CM before the addition of 5 *μ*M A*β*_1-42_ for the next 24 h. Preliminary experiments were carried out with increasing concentrations of A*β* to choose the respective half-maximal inhibitory concentrations—IC50.

### 2.4. Cell Viability and Cytotoxicity Assay

Cell viability was based on the ability of viable cells to convert soluble MTT [3-(4,5n-dimethylthiazol-2-yl)-2-5-diphenyltetrazolium bromide] (Sigma Aldrich) into an insoluble purple formazan. Cells were seeded in 96-well plates. Briefly, cells grown and differentiated as described above were incubated for 4 h at 37°C with MTT (0.5 mg/mL in Neurobasal medium without phenol red). Then, the MTT solution was removed and water-insoluble formazan was immediately dissolved in DMSO (100 *μ*L per well). The amount of formazan was measured at 570 nm with SpectraMax iD3 Multi-Mode Microplate Reader with a version of SoftMaxPro7.1Setup Software (Molecular Devices, LLC., San Jose, CA, USA).

Cytotoxicity was determined by measuring the release of LDH using the Pierce LDH Cytotoxicity Assay Kit (Thermo Fisher Scientific) according to the manufacturer's instructions. The absorbance was measured at 490 nm and 680 nm by SpectraMax iD3 Multi-Mode Microplate Reader with a version of SoftMaxPro7.1Setup Software (Molecular Devices, LLC.). To determine LDH activity, we subtracted the 680 nm absorbance value (background) from the 490 nm absorbance value before calculation of % cytotoxicity [(LDH at 490 nm)—(LDH at 680 nm)]. The percentage of the LDH release was normalized to the condition with the least amount of cell death and divided by a maximum lysis control.

### 2.5. Hoechst Dye 33342 and Propidium Iodide (PI) Double Staining

The SH-SY5Y cells were seeded onto 24-well culture plates (Corning-Costar, Sigma Aldrich, Saint Louis, MO, USA) and were differentiated as described above. On the day 6th, the SH-SY5Y cells (differentiated) were pretreated for 1 h with CM or ALA-CM before the addition of 5 *μ*M A*β*_1-42_ for the next 24 h. Following 24 h incubation, the SH-SY5Y cells were subjected to vital double staining with PI/Hoechst 33342 and evaluated for nuclear morphological changes. Briefly, the SH-SY5Y cells were incubated for 30 min at 37°C (humidified 5% CO_2_/95% air incubator) with PI (1 *μ*g/mL) and HO33342 (5 *μ*g/mL) (Thermo Fisher Scientific), both dissolved in Neurobasal Medium without phenol red. Before measurement, the staining medium was replaced with fresh Neurobasal Medium. Hoechst 33342 is a cell-permeant nuclear counterstain emitting blue fluorescence when bound to DNA, and PI is a membrane impermeant nuclear dye that emits red fluorescence in dead cells, as previously described [17]. The fluorescence of stained SH-SY5Y cells was measured using excitation/emission wavelengths for Hoechst 33342 350/461 nm and 535/617 nm for PI, respectively, by SpectraMax iD3 Multi-Mode Microplate Reader with SoftMaxPro7.1Setup Software (Molecular Devices, LLC.). The stained cells were also analysed in inverted fluorescence microscopy (Olympus IX73, Japan).

### 2.6. Mitochondrial Membrane Potential (*ΔΨ*m)

Mitochondrial depolarization was evaluated using JC-1 Mitochondrial Membrane Potential Assay Kit (Cayman Chemical Company, Ann Arbor, Michigan USA) according to the manufacturer's protocol. The SH-SY5Y cells were seeded in 96-well black plates (Corning-Costar, Sigma Aldrich) and were differentiated as above. On the day 6th, the SH-SY5Y cells (differentiated) were pretreated for 1 h with CM or ALA-CM before the addition of 5 *μ*M A*β*_1-42_ for the next 24 h. Following incubation for 24 h, 10 *μ*L JC-1 Staining Solution (JC-1 Staining Solution was prepared by diluting the reagent 1 : 10 in the culture medium used to culture) was added to each well, and the cells were cultured in the CO_2_ incubator for 30 min at 37°C. Then, the plate was centrifuged for 5 min at 400 g at room temperature. The supernatant was removed, and the plate was rinsed with assay buffer and centrifuged again (the cycle was repeated 5 times). Properly, in healthy cells, JC-1 forms J-aggregates, which display strong fluorescence intensity with excitation and emission at 535 nm and 595 nm, respectively. In apoptotic or unhealthy cells, JC-1 exists as a monomer, which shows a strong fluorescence intensity with excitation and emission at 485 nm and 535 nm, respectively. Fluorescence of J-aggregates and J-monomers was measured using excitation/emission wavelengths of 535/595 nm and 485/535 nm, respectively, by SpectraMax iD3 Multi-Mode Microplate Reader with SoftMaxPro7.1Setup Software (Molecular Devices, LLC.) and inverted fluorescence microscopy (Olympus IX73). The ratio of fluorescence intensity of J-aggregates to the fluorescence intensity of monomers was used to measure the *ΔΨ*m of the SH-SY5Y cells. Mitochondrial depolarization was indicated by an increase in the proportion of cells emitting green fluorescence.

### 2.7. Immunocytofluorescence

The SH-SY5Y cells were seeded onto 8-chamber slides (0.8 cm^2^/well; Lab-Tek, Thermo Fisher Scientific) and were differentiated as described above. On the day 6th, the SH-SY5Y cells (differentiated) were pretreated for 1 h with CM or ALA-CM before the addition of 5 *μ*M A*β*_1-42_ for the next 24 h. Following 24 h incubation, the SH-SY5Y cells were fixed (4% paraformaldehyde, 15 min, RT) and permeabilized by subsequent incubation with 0.5% Triton X-100 (10 min, RT) and nonspecific sites blocked with 1% BSA and 5% normal donkey serum (NDS; 30 min, RT). Next, the cells were incubated with mouse monoclonal anti-*β*_3_ Tubulin antibody (TUJ 1; Santa Cruz Biotechnology, Inc.; 1:  50 in 1% BSA-PBS, for 1 h, RT), rabbit anti-Synaptophysin (GeneTex, Inc.; 1 : 500 in 1% BSA-PBS, for 1 h, RT), mouse anti-PARKIN (Abcam Inc.; 1 : 200 in 1% BSA-PBS, for 1 h, RT), rabbit anti-TOMM20 antibody (Abcam Inc.; 1 : 500 in 1% BSA-PBS, for 1 h, RT), and Alexa Fluor™488-conjugated donkey anti-mouse IgG or Alexa Fluor™568-conjugated goat anti-rabbit IgG (Invitrogen, Life Technologies; 1 : 200 in 1% BSA-PBS, for 1 h, RT). The nuclei were visualized by staining with Hoechst 33342 (Thermo Fisher Scientific; 5 *μ*g/mL, 30 min, RT). In all cases, slides were mounted with Mowiol (Calbiochem-Novabiochem Co. La Jolla, CA, USA) and fluorescence was evaluated in the fluorescence microscope (Olympus IX73). Quantification of fluorescent intensity of TUJ 1, Synaptophysin, TOMM20, and PARKIN in untreated and treated cells was calculated according to the following formula:

Fluorescence intensity = Integrated Density–(Sum area of selected cells x Mean fluorescence of background readings).

Fluorescence intensity was quantitated using the cellSens Entry Version 1 software platform (Olympus Camera, Japan) and Fiji (ImageJ) open-source image processing package [20].

### 2.8. Assessment of Insulin and Insulin-Like Growth Factor I by Enzyme-Linked Immunosorbent Assay

NHA cell culture supernatants were collected and centrifuged at 10 000 g, 4°C for 5 min to remove cell debris. Levels of secreted insulin and IGF-I in cell culture supernatants were measured using a commercially available ELISA kit, (Sigma Aldrich) according to the manufacturer's instructions.

### 2.9. Detection and Quantification of Autophagic Cells by Vital Staining with Acridine Orange

The SH-SY5Y cells were seeded onto 24-well culture plates (Corning-Costar, Sigma Aldrich) and differentiated as mentioned above. On the day 6th, the SH-SY5Y cells (differentiated) were pretreated for 1 h with CM or ALA-CM before the addition of 5 *μ*M A*β*_1-42_ for the next 24 h. Following the 24 h incubation, the SH-SY5Y cells were subjected to vital staining with acridine orange (AO). The SH-SY5Y cells were incubated with AO (1 *μ*g/mL) for 10 min. Before measurement, the staining medium was replaced with fresh Neurobasal Medium without phenol red and analysed in the Olympus IX-73 inverted fluorescence microscope. Quantification of acidic vesicular organelles (AVOs) was calculated as red to green fluorescence intensity ratio (R/G-FIR) in each microscopic field, as described previously [18, 19]. At least 10 replicates for each treatment as well as untreated control cells were quantitated using the cellSens Entry Version 1 software platform (Olympus Camera) and Fiji (ImageJ) open-source image processing package [20].

### 2.10. Quantitative Reverse Transcriptase PCR (RT-qPCR)

Total RNA was extracted from NHA and the SH-SY5Y cells using the Universal RNA Purification Kit (EURX, Poland) according to the manufacturer's protocol. One microgram of total RNA was reverse transcribed to cDNA using High Capacity cDNA Reverse Transcription Kit with RNase Inhibitor (Invitrogen, Life Technologies). For RT-qPCR, the quantification of expression changes was performed 5x HOT FIREPol Eva Green qPCR Mix Plus (Solis BioDyne, Tartu, Estonia) and CFX Connect (Bio-Rad, Hercules, California, USA). Data were normalized to the housekeeping gene and displayed as fold-change compared to the control (DM) using the 2^−*ΔΔ*Ct^ method. Primers are listed in Table 1.

### 2.11. Statistical Analysis

Data from at least three independent experiments were expressed as the mean ± standard error (SEM). Statistical analyses were performed using the one-way analysis of variance (ANOVA) followed by Tukey's multiple comparison post-test and with *P* value being adjusted for multiple comparisons. *P* values of less than 0.05 were considered statistically significant. Statistical differences between the treated cells and untreated control cells were indicated by asterisks (^∗^ for *P* < 0.05; ^∗∗^ for *P* < 0.01; ^∗∗∗^ for *P* < 0.001; ^#^^∗∗∗^ versus the control group). Statistical analyses were performed using GraphPad PrismTM version 5.0 software (GraphPad Software Inc., San Diego, CA, USA).

## 3. Results

### 3.1. Effect of Alpha-Linolenic Acid on the Viability and Secretory Activity of the NHA Cells

**Figure 2:**
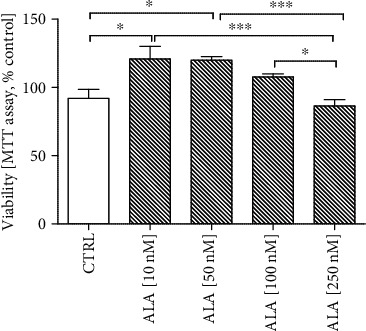
Effect of Alpha-linolenic acid (ALA) on the viability of the Normal Human Astrocytes (NHA). The viability of the NHA cells was increased in 10 nM and 50 nM ALA treatments. The NHA cells were exposed for 24 h to ALA at different doses (10 nM, 50 nM, 100 nM, and 250 nM). The obtained results are presented as a percentage of the control value. One-way ANOVA test for viability followed by Tukey's multiple comparisons was used to analyse the data. Results are presented as means ± SEM (*n* = 6 − 12). Statistical differences between the treated cells and untreated control cells are indicated by asterisks (^∗^ for *P* < 0.05; ^∗∗^ for *P* < 0.01; ^∗∗∗^ for *P* < 0.001).

To determine the optimal dose of ALA, which would stimulate astrocytes to secrete insulin and IGF-I, the NHA cells were treated for 24 h with ALA at different doses (10 nM, 50 nM, 100 nM, and 250 nM). First, we checked the effect of ALA on the viability of the NHA cells by MTT assay. As illustrated in Figure 2, 10 nM and 50 nM ALA treatments increased the NHA cells viability to 120% compared with the control group (100%; *P* < 0.05). When the dose of ALA was increased to 250 nM, the viability decreased to 86% (Figure 2).

**Figure 3:**
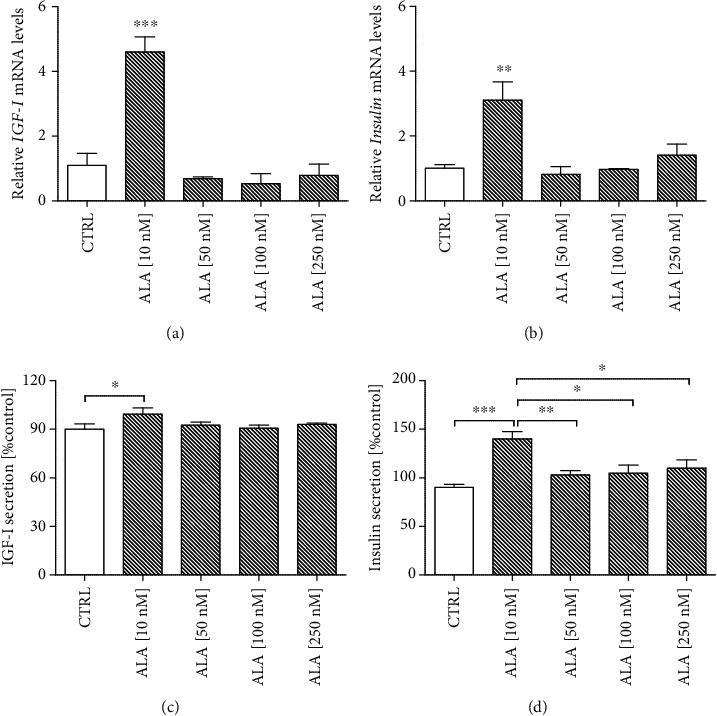
Effect of Alpha-linolenic acid (ALA) on mRNA and protein expression of Insulin and Insulin-Like Growth Factor I (IGF-I). Quantitative reverse transcriptase PCR (RT-qPCR) results indicated that 10 nM ALA treatment significantly increased the mRNA expression of IGF-I and Insulin in the NHA cells (a, b). Moreover, the ELISA analysis showed that 10 nM ALA significantly increased the release of IGF-I and insulin from the NHA cells to the medium (c, d). The NHA cells were exposed for 24 h to ALA at different doses (10 nM, 50 nM, 100 nM, and 250 nM). One-way ANOVA followed by Tukey's multiple comparisons test at the 0.05 level was used to determine differences between treated cells and untreated control cells. Results are presented as means ± SEM (*n* = 3 − 8). RT-qPCR fold increase was calculated according to the formula described in the Materials and Methods section. Statistical differences between treated cells and untreated control cells are indicated by asterisks (^∗^ for *P* < 0.05; ^∗∗^ for *P* < 0.01; ^∗∗∗^ for *P* < 0.001).

Then, we assessed by RT-qPCR the effect of different doses of ALA on IGF-I and insulin mRNA production in the NHA cells (Figures 3(a) and 3(b)). We found that 10 nM ALA treatment significantly increased the mRNA expression of *IGF-I* (Figure 3(a); *P* < 0.001) and insulin (Figure 3(b); *P* < 0.01). Likewise, 10 nM ALA treatment-induced astrocytes to secrete IGF-I (Figure 3(c); *P* < 0.05) and insulin (Figure 3(d); *P* < 0.001) into the culture medium.

Based on the above results, a dose of 10 nM ALA was used for the experiments.

### 3.2. Effect of CM and ALA-CM on Cell Viability of Differentiated SH-SY5Y Cells

We hypothesized that compound secreted by astrocytes might exert protective effects against A*β*-induced cytotoxicity. To investigate the potential neuroprotective effects of CM and ALA-CM, differentiated SH-SY5Y cells were pretreated with CM and ALA-CM for 1 h before A*β*_1-42_ treatment.

**Figure 4:**
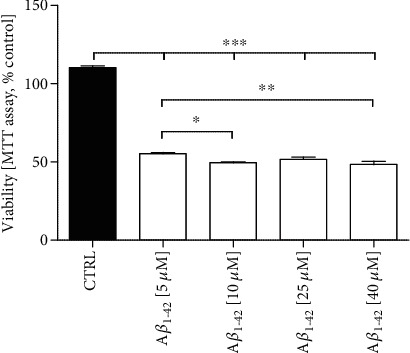
Effect of Amyloid *β*_1-42_ (A*β*_1-42_) treatment on differentiated SH-SY5Y cell viability. A*β*_1-42_ treatment significantly inhibited the cell viability of differentiated SH-SY5Y cells in a dose-dependent manner as compared with the control. Differentiated SH-SY5Y cells (on the day 6th) were exposed for 24 h to A*β*_1-42_ at different doses (5 *μ*M, 10 *μ*M, 25 *μ*M, 40 *μ*M). The obtained results are presented as a percentage of the control value. One-way ANOVA test for viability followed by Tukey's multiple comparisons was used to analyse the data. Results are presented as means ± SEM (*n* = 7 − 8). Statistical differences between the treated and untreated control cells are indicated by asterisks (^∗^ for *P* < 0.05; ^∗∗^ for *P* < 0.01; ^∗∗∗^ for *P* < 0.001).

We observed that A*β*_1-42_ treatment effectively inhibited the cell viability of differentiated SH-SY5Y cells in a dose-dependent manner as compared with the control (Figure 4; *P* < 0.001). When the SH-SY5Y cells were exposed to 5 *μ*M of A*β*_1-42_ for 24 hours, cell viability was reduced to approximately 50% of the control (Figure 4; *P* < 0.001). Thus, the concentration of 5 *μ*M of A*β*_1-42_ was used for further investigations.

**Figure 5:**
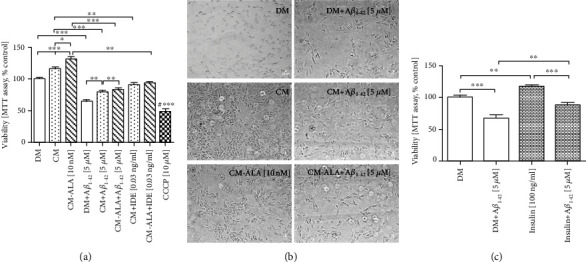
CM and ALA-CM reversed the effects of Amyloid *β*_1-42_ (A*β*_1-42_) treatment on differentiated SH-SY5Y cell viability. The results showed that the CM and ALA-CM pretreatment significantly increased the viability of differentiated SH-SY5Y cells and restored A*β*_1-42_-induced reduction of the cell viability (a). Insulin Degrading Enzyme (IDE) treatment of CM and ALA-CM reduced this effect. On the day 6th, the SH-SY5Y cells (differentiated) were pretreated for 1 h with CM or ALA-CM before the addition of 5 *μ*M A*β*_1-42_ for the next 24 h. The SH-SY5Y cells were also exposed to cotreatment of CM and ALA-CM with IDE to check whether insulin and IGF-I presence in CM and ALA-CM was responsible for the neuroprotective effect. Positive controls were the SH-SY5Y cells treated with insulin (c) and carbonyl-cyano-m-chlorophenylhydrazone-CCCP (10 *μ*M). The obtained results are presented as a percentage of the control value. One-way ANOVA test for viability followed by Tukey's multiple comparisons was used to analyse data. Statistical differences between the treated cells and untreated control cells are indicated by asterisks (^∗^ for *P* < 0.05; ^∗∗^ for *P* < 0.01; ^∗∗∗^ for *P* < 0.001; ^#^^∗∗∗^ versus the control group). Results are means ± SEM of three independent experiments. Cell morphology was observed under a microscope (b). Scale bar is 20 *μ*m.

As shown on Figure 5, the CM pretreatment significantly increased the cell viability (*P* < 0.001), and restored the cell viability (to 79.16%) compared to control system (64%; DM + A*β*_1−42_) (*P* < 0.01). Moreover, the ALA-CM pretreatment intensified the neuroprotective effect of CM (Figure 5; *P* < 0.01). A similar effect was observed after treatment of the SH-SY5Y cells with insulin (Figure 5(c); *P* < 0.01). Additionally, the insulin pretreatment significantly increased the SH-SY5Y cell viability compared to the control model (DM + A*β*_1−42_) (Figure 5(c); *P* < 0.01).

Next, to check whether insulin and IGF-I presence in CM and ALA-CM was responsible for the neuroprotective effect, we treated CM and ALA-CM with the insulin-degrading enzyme (IDE), which degrades both insulin and IGF-I. Cotreatment of CM and ALA-CM with IDE significantly decreased the cell viability to 90.53% and 93.3%, compared to CM and ALA-CM groups, respectively (Figure 5; *P* < 0.01).

### 3.3. The CM and ALA-CM Pretreatment Reversed the A*β*_1-42_-Induced Cytotoxicity in Differentiated SH-SY5Y Cells

The inhibitory effects of CM and ALA-CM on A*β*_1-42_–induced cytotoxicity were evaluated by measuring LDH levels and double-stained with Hoechst 33342 and propidium iodide.

**Figure 6:**
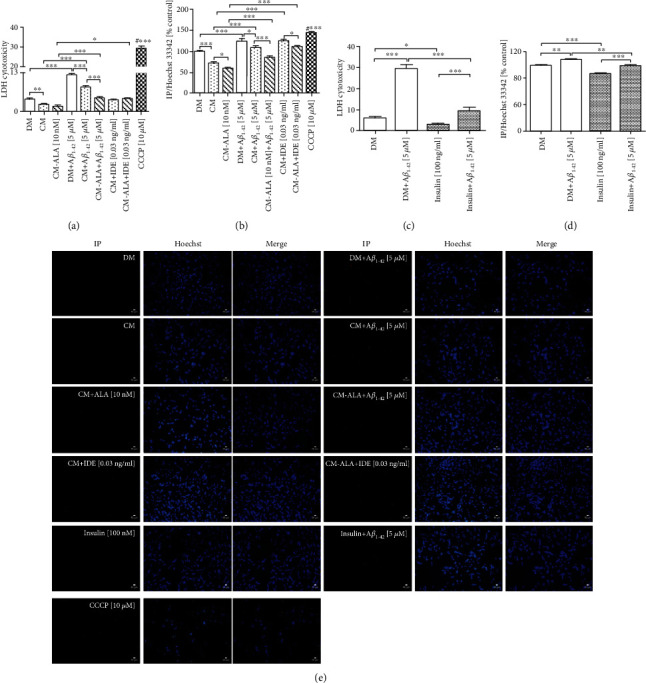
The CM and ALA-CM pretreatment reversed the Amyloid *β*_1-42_- (A*β*_1-42_-) induced cytotoxicity in differentiated SH-SY5Y cells. The LDH assay (a, c) and double-stained with Hoechst 33342 and propidium iodide (PI) (b, d, e) results indicated that A*β*_1-42_ significantly increased cell death of differentiated SH-SY5Y cells. Whereas, the CM pretreatment decreased cell death in the SH-SY5Y cells treated with A*β*_1-42_. Besides, ALA preactivated CM significantly intensified the protective effect of CM. In contrast, Insulin Degrading Enzyme (IDE) significantly attenuated the protective effect of CM and ALA-CM. The insulin pretreatment reversed the A*β*_1-42_-induced cytotoxicity in differentiated SH-SY5Y cells. On the day 6th, the SH-SY5Y cells (differentiated) were pretreated for 1 h with CM or ALA-CM before the addition of 5 *μ*M A*β*_1-42_ for the next 24 h. The SH-SY5Y cells were also exposed to cotreatment of CM and ALA-CM with IDE to check whether insulin and IGF-I presence in CM and ALA-CM was responsible for the neuroprotective effect. Positive controls were the SH-SY5Y cells treated with Insulin and carbonyl-cyano-m-chlorophenylhydrazone-CCCP (10 *μ*M). Next, cells were subjected to vital double staining with PI and Hoechst 33342 (see Materials and Methods section). The PI/Hoechst ratio was calculated by dividing the PI by Hoechst Relative fluorescence units (RFUs). One-way ANOVA test for cytotoxicity followed by Tukey's multiple comparisons was used to analyse data. Statistical differences between the treated cells and untreated control cells are indicated by asterisks (^∗^ for *P* < 0.05; ^∗∗^ for *P* < 0.01; ^∗∗∗^ for *P* < 0.001; ^#^^∗∗∗^ versus the control group). Results are means ± SEM of three independent experiments. The vital double staining cells were also analyzed in inverted fluorescence microscopy (see Materials and Methods section). Scale bar is 50 *μ*m.

Differentiated SH-SY5Y cells treated with A*β*_1-42_ for 24 h expressed 12.25% LDH release as compared to the control group (4%) (Figure 6(a); *P* < 0.001). However, the CM and ALA-CM pretreatment attenuated LDH activity. The CM-pretreatment markedly decreased LDH release to 8.08% (Figure 6(a); *P* < 0.001). In addition, the ALA-CM pretreatment showed a stronger cytoprotective effect than CM as LDH release was decreased to 4.6% (Figure 6(a); *P* < 0.001).

Similar to the LDH release results, the cytoprotective effect of CM and ALA-CM was confirmed by using double-stained with Hoechst 33342 and propidium iodide (Figures 6(a) and 6(b)). Data indicated that A*β*_1-42_ treated cells (DM + A*β*_1−42_) had a significantly increased (*P* < 0.001) ratio of PI/Hoechst fluorescence signal (Figure 6(b)), which resulted from the increase in cell death. The CM and ALA-CM pretreatment decreased (*P* < 0.001) the ratio of PI/Hoechst fluorescence signal as compared to the control group (Figure 6(b)). Moreover, the CM pretreatment decreased cell death in the SH-SY5Y cells treated with A*β*_1-42_ when compared with DM + A*β*_1−42_ group (Figure 6(b); *P* < 0.05). Interestingly, ALA preactivated CM significantly intensified the protective effect of CM (Figure 6(b); *P* < 0.001). In contrast, IDE significantly attenuated the protective effect of CM and ALA-CM (Figures 6(a) and 6(b), *P* < 0.001).

A similar effect was observed after the treatment of differentiated SH-SY5Y cells with insulin (Figure 6(c); *P* < 0.05; Figure 6(d); *P* < 0.001, Figure 6(e)). Moreover, the insulin pretreatment reversed the A*β*_1-42_-induced cytotoxicity in differentiated SH-SY5Y cells. (Figure 6(c); *P* < 0.001, Figure 6(d); *P* < 0.01, Figure 6(c)).

These results indicated that astrocyte-derived insulin/insulin-like growth factor I protect differentiated SH-SY5Y cells against A*β*_1-42_-induced cell death.

### 3.4. The CM and ALA-CM Pretreatment Reversed the A*β*_1-42_-Induced Synaptic Toxicity in Differentiated SH-SY5Y Cells

**Figure 7:**
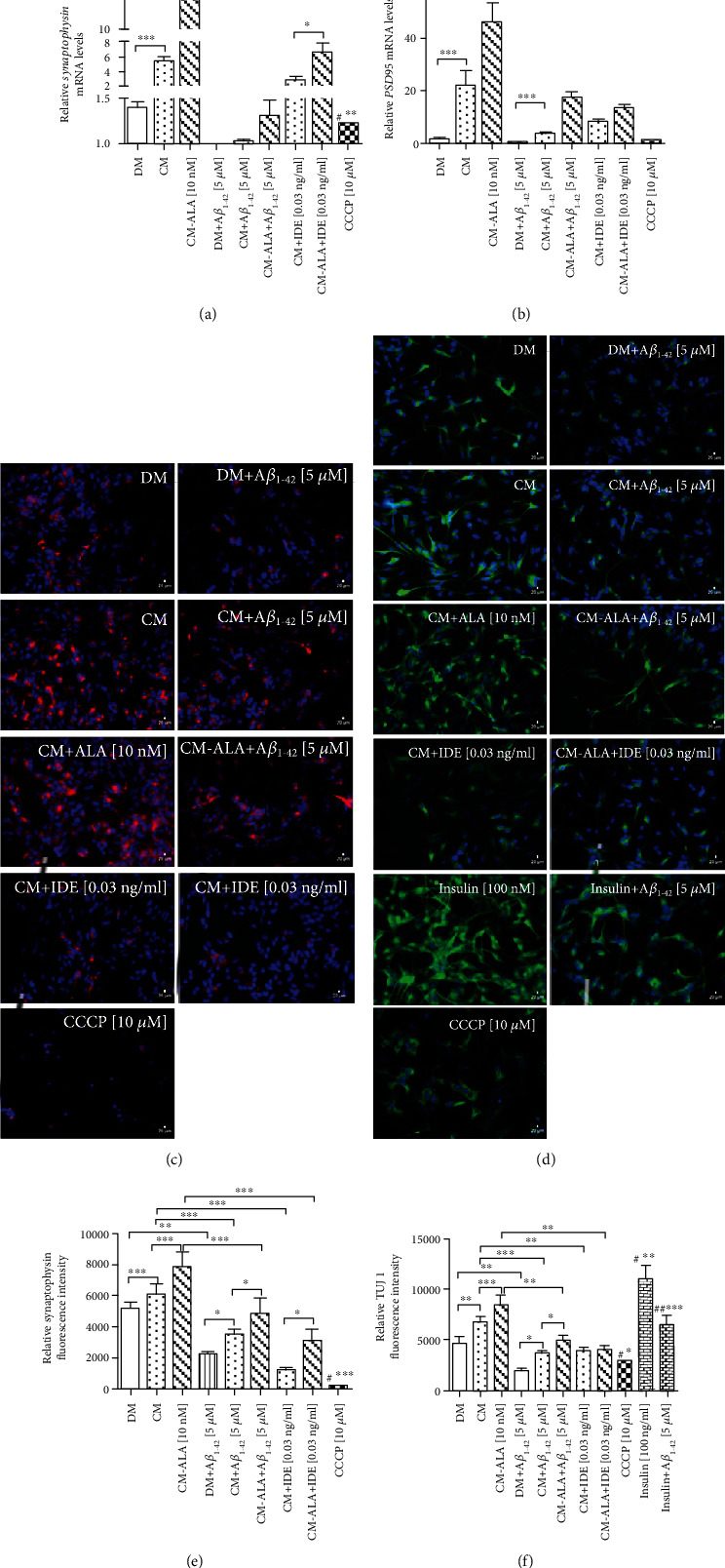
The CM and ALA-CM pretreatment reversed Amyloid *β*- (A*β*_1-42_-) induced synaptic toxicity in differentiated SH-SY5Y cells. On the day 6th, the SH-SY5Y cells (differentiated) were pretreated for 1 h with CM or ALA-CM before the addition of 5 *μ*M A*β*_1-42_ for the next 24 h. The SH-SY5Y cells were also exposed to the cotreatment of CM and ALA-CM with Insulin Degrading Enzyme (IDE) to check whether insulin and IGF-I presence in CM and ALA-CM was responsible for the neuroprotective effect. Positive controls were the SH-SY5Y cells treated with insulin and carbonyl-cyano-m-chlorophenylhydrazone-CCCP (10 *μ*M). RT-qPCR results indicated that A*β*_1-42_ significantly decreased mRNA levels of *Synaptophysin* (a) and *PSD95* (b), well-known synaptic markers. The CM and ALA-CM pretreatment reversed the effect of A*β*_1-42_ when compared with DM + A*β*_1−42_ group. The IDE treatment of CM and ALA-CM reduced this effect. The immunocytofluorescence staining showed that the A*β*_1-42_ treated cells had a decreased Synaptophysin (c, e) and TUJ 1 (*β*3-Tubulin) (d, f) fluorescence intensity and increased neurites fragmentation. Moreover, results showed that the CM and ALA-CM pretreatment reversed the A*β*_1-42_–induced synaptic toxicity in differentiated SH-SY5Y cells. A similar effect of TUJ 1 fluorescence intensity was observed after the treatment of differentiated SH-SY5Y cells with insulin (d, f). The IDE treatment of CM and ALA-CM reduced this effect. The cells were subjected to immunocytofluorescence staining with antibodies against Synaptophysin and TUJ 1. TUJ 1 was used as a marker to stain differentiated SH-SY5Y cells (show as green signals). Synaptophysin was used to stain synaptic in differentiated SH-SY5Y cells (show as red signals). Hoechst 33342 was used to stain nuclei (show as blue signals) (see Materials and Methods section). Bar graphs (e, f) showed the relative fluorescence intensity of Synaptophysin and TUJ 1. Scale bar is 20 *μ*m. One-way ANOVA followed by Tukey's multiple comparisons test at the 0.05 level was used to determine differences between the treated cells and untreated control cells. Results are presented as means ± SEM (*n* = 3 − 8). RT-qPCR fold increase and the fluorescence intensity were calculated according to the formula described in the Materials and Methods section. Statistical differences between the treated group and untreated control cells are indicated by asterisks (^∗^ for *P* < 0.05; ^∗∗^ for *P* < 0.01; ^∗∗∗^ for *P* < 0.001; ^#^^∗∗∗^ versus the control group; ^##^^∗∗∗^ versus DM + A*β*_1−42_ group).

To elucidate the effect of CM and ALA-CM on A*β*_1-42_-induced synaptic toxicity in differentiated SH-SY5Y cells, we measured the expression of synaptic markers through RT-qPCR and immunocytofluorescence methods. RT-qPCR results indicated that pretreatment with CM increased mRNA levels of *Synaptophysin* and *PSD95* (Figures 7(a) and 7(b); *P* < 0.001) in comparison to the control group. Moreover, ALA preactivated CM significantly intensified this effect (*P* < 0.001). However, the A*β*_1-42_ treatment markedly decreased mRNA levels of *Synaptophysin* (Figure 7(a); *P* < 0.001) and *PSD95* (Figure 7(b); *P* < 0.05) when compared to the control group. In contrast, the CM and ALA-CM pretreatment increased levels of *PSD95* in the SH-SY5Y cells treated with A*β*_1-42_ as compared with DM + A*β*_1−42_ group (Figure 7(b); *P* < 0.001). Cotreatment of CM and ALA-CM with IDE significantly attenuated their effect on mRNA expression of synaptic markers.

Further evaluation of the effect of CM and ALA-CM on A*β*_1-42_-induced synaptic toxicity and neuronal phenotype of differentiated SH-SY5Y cells was established by using immunocytofluorescence staining with antibodies against Synaptophysin and microtubule component *β*_3_-tubulin TUJ 1 (Figures 7(c)–7(f)). Similar to RT-qPCR results, the A*β*_1-42_ treated cells (DM + A*β*_1−42_) had a decreased Synaptophysin (Figures 7(c) and 7(e); *P* < 0.001) and TUJ 1 (Figures 7(d) and 7(f); *P* < 0.001) fluorescence intensity. Moreover, the A*β*_1-42_ treatment induced fragmentation of neuritis (Figure 7(d)). In contrast, the CM and ALA-CM pre-treatment reversed A*β*_1-42_–induced synaptic toxicity in differentiated SH-SY5Y cells (Figures 7(c)–7(f); *P* < 0.05). Moreover, the insulin pretreatment reversed the A*β*_1-42_-induced decrease of TUJ 1 fluorescence intensity and fragmentation of neuritis in differentiated SH-SY5Y cells (Figures 7(d) and 7(f); *P* < 0.001). Cotreatment of CM and ALA-CM with IDE attenuated these effects.

### 3.5. The CM and ALA-CM Pretreatment Protected the SH-SY5Y Cells against A*β*_1-42_-Induced Mitochondrial Dysfunction

**Figure 8:**
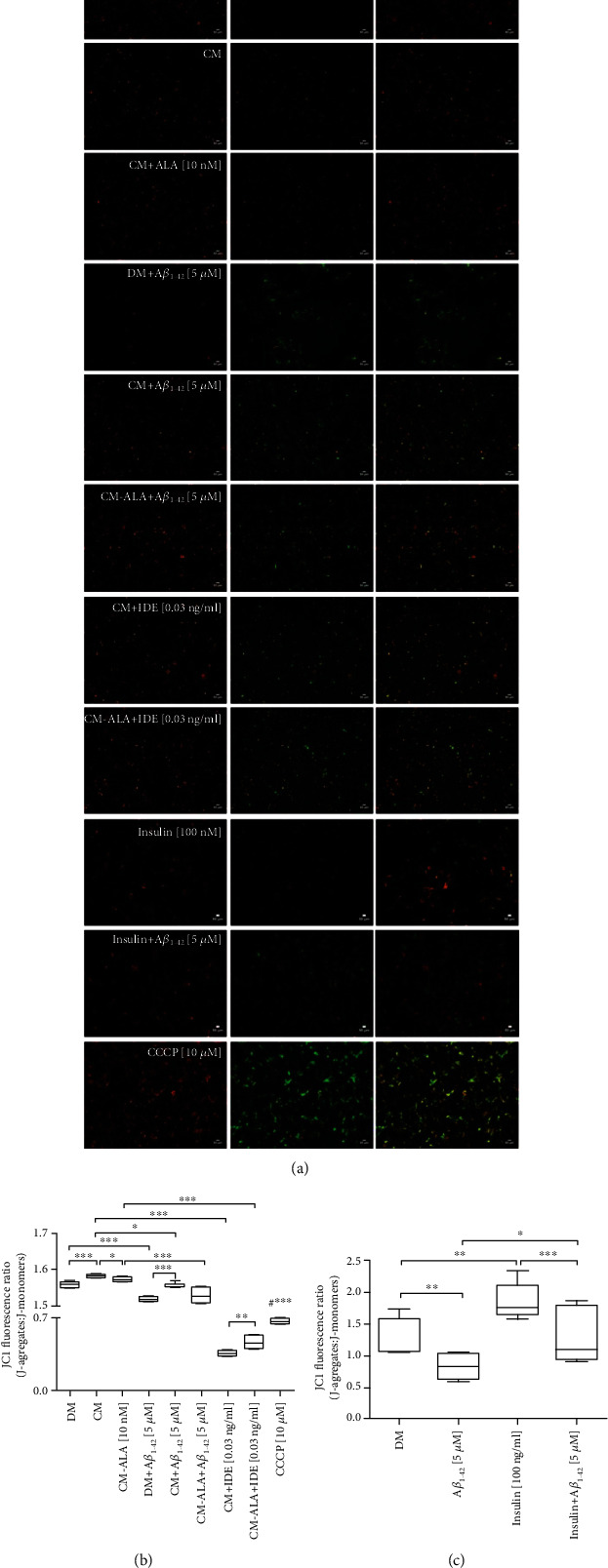
The CM and ALA-CM pretreatment inhibits Amyloid *β*- (A*β*_1-42_-) induced depolarization of the mitochondrial membrane in differentiated SH-SY5Y cells. Representative fluorescence microscopy images of 5,5,6,6′-tetrachloro-1,1′,3,3′ tetraethylbenzimi-dazoylcarbocyanine iodide (JC-1) staining (a) and the ratio of fluorescence intensity of J-aggregates to the fluorescence intensity of monomers (b, c) was used to measure mitochondrial membrane potential (*ΔΨ*m) of differentiated SH-SY5Y cells. Results showed that A*β*_1-42_ treatment decreased the *ΔΨm* of differentiated SH-SY5Y cells. The CM and ALA-CM pretreatment reversed the effect of A*β*_1-42_ compared with DM + A*β*_1−42_ group. The Insulin Degrading Enzyme (IDE) treatment of CM and ALA-CM reduced this effect. On the day 6th, the SH-SY5Y cells (differentiated) were pretreated for 1 h with CM or ALA-CM before the addition of 5 *μ*M A*β*_1-42_ for the next 24 h. The SH-SY5Y cells were also exposed to cotreatment of CM and ALA-CM with IDE to check whether insulin and IGF-I presence in CM and ALA-CM was responsible for the neuroprotective effect. Carbonyl cyanide 3-chlorophenylhydrazone (CCCP) was used as a mitochondrial membrane potential disruptor. Insulin was used as a positive control. Next, cells were subjected to JC-1 staining (see Materials and Methods section). Fluorescence of JC-1 was measured by a fluorescence microscope and microplate reader. One-way ANOVA followed by Tukey's multiple comparisons test at the 0.05 level was used to determine differences between the treated cell and untreated control cells. Results are presented as means ± SEM (*n* = 4 − 6). The ratio of fluorescence intensity of J-aggregates (shown as red signals) to the fluorescence intensity of monomers (shown as green signals) was calculated according to the formula described in the Materials and Methods section. Statistical differences between the treated cells and untreated control cells are indicated by asterisks (^∗^ for *P* < 0.05; ^∗∗^ for *P* < 0.01; ^∗∗∗^ for *P* < 0.001; ^#^^∗∗∗^ versus the control group). Scale bar is 50 *μ*m.

**Figure 9:**
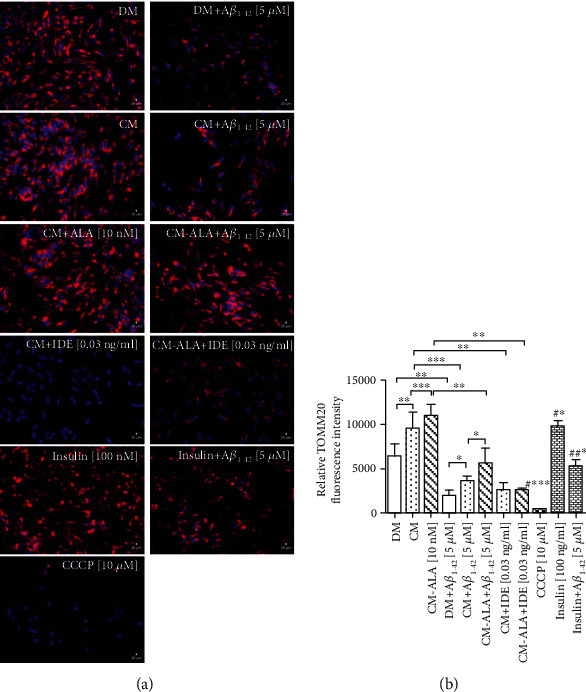
The CM and ALA-CM pretreatment reversed Amyloid *β* (A*β*1-42) induced a reduction in mitochondrial mass in differentiated SH-SY5Y cells. The fluorescence intensity indicating mitochondrial mass was calculated by immunocytofluorescence staining of the translocase of outer mitochondrial membrane 20 (TOMM20) in differentiated SH-SY5Y cells. Representative fluorescence images (a) and the relative fluorescence intensity of TOMM20 (b) showed that A*β*1-42 induced a reduction of TOMM20 fluorescence intensity. However, the decrease in TOMM20 fluorescence intensity was improved by the CM pretreatment of differentiated SH-SY5Y cells before A*β*1-42 exposure. Besides, ALA-preactivated CM intensified this effect. In contrast, cotreatment of CM and ALA-CM with Insulin Degrading Enzyme (IDE) markedly decreased the immunoreactivity of TOMM20-positive mitochondrial. On the day 6th, the SH-SY5Y cells (differentiated) were pretreated for 1 h with CM or ALA-CM before the addition of 5 *μ*M A*β*1-42 for the next 24 h. The SH-SY5Y cells were also exposed to cotreatment of CM and ALA-CM with IDE to check whether insulin and IGF-I presence in CM and ALA-CM was responsible for the neuroprotective effect. Carbonyl cyanide 3-chlorophenylhydrazone (CCCP) was used as a mitochondrial membrane potential disruptor. Insulin was used as a positive control. Next, cells were subjected to immunocytofluorescence staining with antibodies against TOMM20 (see Materials and Methods section). TOMM20 was used to stain mitochondria in differentiated SH-SY5Y cells (shown as red signals). Hoechst 33342 was used to stain nuclei (shown as blue signals). Bar graph showed the relative fluorescence intensity of TOMM20. The fluorescence intensity of TOMM20 was calculated according to the formula described in the Materials and Methods section. Statistical differences between the treated cells and untreated control cells are indicated by asterisks (^∗^ for *p* < 0.05; ^∗∗^ for *p* < 0.01; ^∗∗∗^ for *p* < 0.001; #^∗∗∗^, #^∗^ versus the control group; ##^∗^; versus DM + A*β*1-42 group). Scale bar is 20 *μ*m.

Mitochondrial dysfunction is proposed to participate in cellular apoptosis. Depolarization of the mitochondrial membrane is a sensitive indicator of mitochondrial function. Therefore, the *Δψ*m in the SH-SY5Y cells was evaluated by detecting the red/green fluorescence intensity ratio of JC-1 staining (Figures 8(a)–8(c) and 9(b)). The positive control was the SH-SY5Y cells treated with carbonyl-cyano-m-chlorophenylhydrazone-CCCP (10 *μ*M). CCCP causes uncoupling of the electron transport of the respiratory chain, reducing the mitochondrial membrane potential, which consequently induces cell death. As shown in Figures 8(a) and 8(b), treatment with A*β*_1-42_ significantly increased green fluorescence intensity in the SH-SY5Y cells (*P* < 0.001). The A*β*_1-42_ exposure has a similar effect to CCCP suggesting that A*β*_1-42_ significantly reduced *Δψ*m. Whereas pretreatment with CM and ALA-CM remarkably reduced green fluorescence intensity and increased red fluorescence intensity in the SH-SY5Y cells treated with A*β*_1-42_ as compared with DM + A*β*_1−42_ treated group (*P* < 0.001). However, cotreatment of CM and ALA-CM with IDE significantly decreased *Δψm* (*P* < 0.001).

Similarly, the insulin treatment increased red fluorescence intensity in differentiated SH-SY5Y cells (Figure 8(c); *P* < 0.01). Additionally, the insulin pretreatment significantly increased *Δψm* when compared to DM + A*β*_1−42_ treated group (Figure 8(c); *P* < 0.05).

This finding indicates that astrocyte-derived insulin/insulin-like growth factor I inhibits A*β*_1-42_-induced depolarization of the mitochondrial membrane in differentiated SH-SY5Y cells.

**Figure 10:**
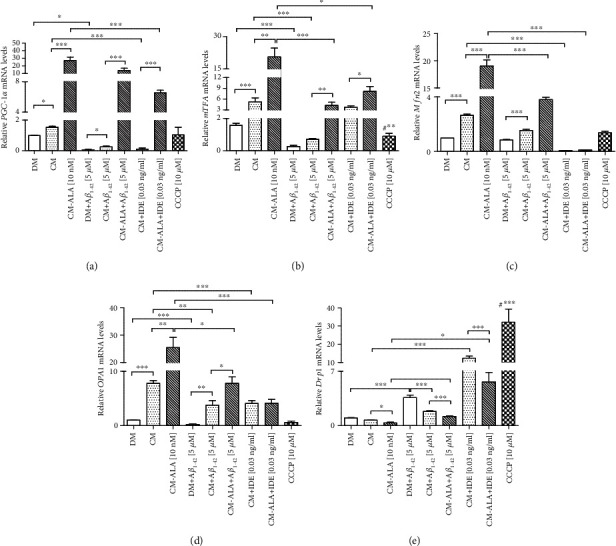
The CM and ALA-CM pretreatment regulated the mitochondrial biogenesis and dynamics in differentiated SH-SY5Y cells. RT-qPCR results indicated that Amyloid *β* (A*β*1-42) significantly decreased mRNA levels of PGC-1*α* (a), mTFA (b), Mfn2 (c), and OPA1 (d), and increased levels of Drp1 (e). Amyloid *β* (A*β*) A*β*1-42-induced reduction on mitochondrial biogenesis was restored by CM and ALA-preactivated CM. Moreover, the CM and ALA-CM pretreatment regulated the balance between fission and fusion processes. Cotreatment of CM and ALA-CM with Insulin Degrading Enzyme (IDE) decreased mRNA expression of genes involved in mitochondrial biogenesis and promoted the elevation of mRNA levels of Drp1, a fission gene. On the day 6th, the SH-SY5Y cells (differentiated) were pretreated for 1 h with CM or ALA-CM before the addition of 5 *μ*M A*β*1-42 for the next 24 h. The SH-SY5Y cells were also exposed to cotreatment of CM and ALA-CM with IDE to check whether insulin and IGF-I presence in CM and ALA-CM was responsible for the neuroprotective effect. The positive control was the SH-SY5Y cells treated with carbonyl-cyano-m-chlorophenylhydrazone-CCCP (10 *μ*M). One-way ANOVA followed by Tukey's multiple comparisons test at the 0.05 level was used to determine differences between the treated cells and untreated control cells. Results are presented as means ± SEM (*n* = 3 − 8). RT-qPCR fold increase was calculated according to the formula described in the Materials and Methods section. Statistical differences between the treated cells and untreated control cells are indicated by asterisks (^∗^ for *p* < 0.05; ^∗∗^ for *p* < 0.01; ^∗∗∗^ for *p* < 0.001; #^∗∗^ versus the control; #^∗∗∗^ versus the control group).

Then, we examined the effect of CM and ALA-CM on mRNA expression of mitochondrial biogenesis and dynamics genes. First, we analysed mRNA levels of the *PGC-1α* gene and its target gene *mTFA*, which is directly involved in mitochondrial biogenesis. As shown in Figures 10(a) and 10(b), treatment with A*β*_1-42_ significantly decreased mRNA transcripts levels of *PGC-1α* (*P* < 0.05) and its downstream target gene *mTFA* (*P* < 0.001). The pretreatment with CM and ALA-CM increased mRNA levels of *PGC-1α* and mTFA (*P* < 0.001) compared to the control. Moreover, pretreatment with CM and ALA-CM reversed the inhibition of *PGC-1α* (*P* < 0.05) and *mTFA* (*P* < 0.001) mRNA expression caused by A*β*_1-42_. Cotreatment of CM and ALA-CM with IDE significantly decreased mRNA expression of genes involved in the mitochondrial biogenesis.

Mitochondria are highly dynamic organelles with morphology and numbers regulated by fission and fusion proteins. Excessive mitochondrial fragmentation leads to impaired mitochondrial function and neuronal death in AD. Therefore, in the next step, we also investigated the regulating signals in mitochondrial fission and fusion processes. Mitofusins 2 (Mfn2) in the outer mitochondrial membrane and optic atrophy 1 (Opa1) in the inner mitochondrial membrane regulate the fusion process, and dynamin-related protein 1 (Drp1) regulates the mitochondrial fission. The SH-SY5Y cells exposed to A*β*_1-42_ showed reduced mRNA transcripts levels of *Mfn2* and *Opa1,* and increased levels of mRNA of *Drp1* (Figures 10(c)–10(e)); *P* < 0.001). Whereas pretreatment with CM and ALA-CM of the SH-SY5Y cells exposed to A*β*_1-42_ rescued the levels of these transcripts to values similar to those of the untreated cells. Also, cotreatment of CM and ALA-CM with IDE significantly increased mRNA expression of *Drp1* (Figure 10(e); *P* < 0.001).

To determine the mitochondrial content in the SH-SY5Y cells, we probed for the mitochondrial outer membrane protein TOMM20 (translocase of outer mitochondrial membrane 20) by using the immunocytofluorescence method. As shown in Figures 9(a) and 9(b), TOMM20 fluorescence intensity was decreased in A*β*_1-42_-treated SH-SY5Y cells (DM + A*β*_1−42_) (*P* < 0.001). Whereas pretreatment with CM of the SH-SY5Y cells exposed to A*β*_1-42_ increased the immunoreactivity of TOMM20-positive mitochondrial in comparison with DM + A*β*_1−42_ group (*P* < 0.05). Furthermore, pretreatment with ALA-CM increased TOMM20 fluorescence intensity in comparison with CM + A*β*_1−42_ group (*P* < 0.05). However, the SH-SY5Y cells exposed to cotreatment of CM and ALA-CM with IDE showed a decrease in fluorescence intensity of TOMM20 when compared to CM and ALA-CM groups (Figures 9(a) and 9(b); *P* < 0.001). Likewise, we observed that the insulin treatment increased TOMM20 fluorescence intensity (Figures 9(a) and 9(b); *P* < 0.05). Similarly, pretreatment with insulin increased TOMM20 fluorescence intensity in comparison with DM + A*β*_1−42_ group (Figures 9(a) and 9(b); *P* < 0.05).

In agreement with the above results, astrocyte-derived insulin/insulin-like growth factor I restored the A*β*_1-42_ -damaged of mitochondrial biogenesis and dynamic processes.

### 3.6. The CM and ALA-CM Pretreatment Modulates A*β*_1-42_-Induced Effects on Mitophagy and Autophagy

**Figure 11:**
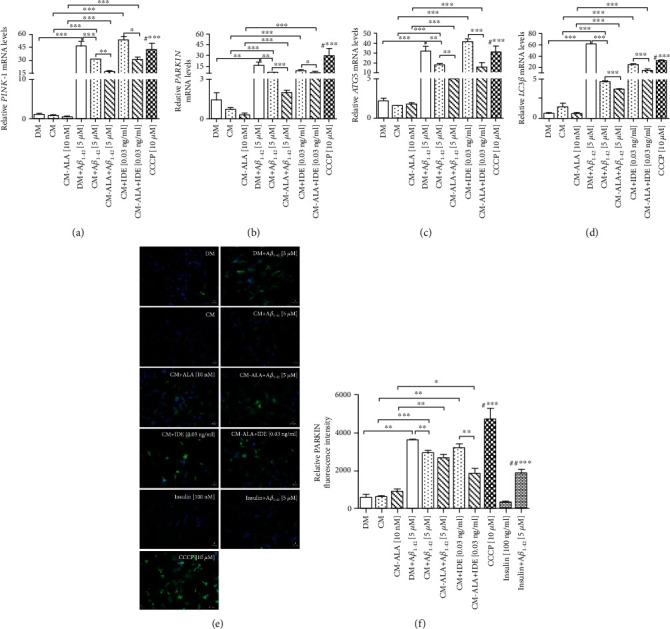
The CM and ALA-CM pretreatment modulates Amyloid *β*- (A*β*_1-42_-) induced effects on mitophagy and autophagy. On the day 6th, the SH-SY5Y cells (differentiated) were pretreated for 1 h with CM or ALA-CM before the addition of 5 *μ*M A*β*1-42 for the next 24 h. The SH-SY5Y cells were also exposed to the cotreatment of CM and ALA-CM with Insulin Degrading Enzyme (IDE) to check whether insulin and IGF-I presence of CM and ALA-CM was responsible for the neuroprotective effect. Positive controls were the SH-SY5Y cells treated with insulin and carbonyl-cyano-m-chlorophenylhydrazone-CCCP (10 *μ*M). RT-qPCR results showed that A*β*_1-42_ significantly increased mRNA levels of markers of mitophagy (*PINK-1* (a), *PARKIN* (b)), and autophagy (*ATG5* (c) and *LC3β* (d)). Whereas pretreatment with CM of the SH-SY5Y cells exposed to A*β*_1-42_ significantly decreased expression of mitophagy and autophagy markers. IDE increased levels of mitophagy and autophagy markers. The immunocytofluorescence staining showed that the A*β*_1-42_ treated cells had an increased PARKIN fluorescence intensity, a well-known marker of mitophagy (e, f). Whereas pretreatment with CM and ALA-CM of the SH-SY5Y cells exposed to A*β*_1-42_ significantly decreased PARKIN fluorescence intensity. A similar effect was observed after the insulin treatment. IDE increased PARKIN fluorescence intensity. Bar graph showed the relative fluorescence intensity of PARKIN. Antibody against PARKIN was used to stain marker of mitophagy in differentiated SH-SY5Y cells (shown as green signals). Hoechst 33342 was used to stain nuclei (shown as blue signals). Scale bar is 20 *μ*m. The results showed that A*β*_1-42_ exposure has a similar effect to CCCP suggesting that A*β*_1-42_ induce mitophagy and autophagy. One-way ANOVA followed by Tukey's multiple comparisons test at the 0.05 level was used to determine differences between the treated cells and untreated control cells. Results are presented as means ± SEM (*n* = 3 − 8). RT-qPCR fold increase and the fluorescence intensity were calculated according to the formula described in the Materials and Methods section. Statistical differences between the treated cells and untreated control cells are indicated by asterisks (^∗^ for *P* < 0.05; ^∗∗^ for *P* < 0.01; ^∗∗∗^ for *P* < 0.001; ^#^^∗∗∗^ versus the control group; ^##^^∗∗∗^ versus DM + A*β*_1−42_ group).

As observed above, decreased levels of mitochondrial profusion genes and increased levels of mitochondrial profission genes may result in autophagic clearance of damaged mitochondria. As CM and ALA-CM rescued the A*β*_1-42_-induced mitochondrial dysfunction, we further investigated these effects on subsequent mitophagy. Autophagy (mitophagy) process has been suggested to remove damaged and dysfunctional mitochondria, which has been implicated in the progression of AD [21]. In this experiment, the potent depolarizing agent CCCP was used as a positive control to chemically induce mitophagy and autophagy. We determined mitophagy by monitoring expression levels of *PINK-1* and *PARKIN*, well-known markers of this process. As shown in Figures 11(a) and 11(b), A*β*_1-42_ treatment markedly increased mRNA levels of *PINK-1* (*P* < 0.001) and *PARKIN* (*P* < 0.01) in comparison to the control group. A*β*_1-42_ exposure had a similar effect to CCCP, suggesting that A*β*_1-42_ significantly increased mitophagy. Whereas pretreatment with CM of the SH-SY5Y cells exposed to A*β*_1-42_ significantly decreased the level of mitophagy markers when compared to DM + A*β*_1−42_ group (Figure 11(a); *P* < 0.001, Figure 11 (b); *P* < 0.01). Interestingly, ALA preactivated CM significantly intensified this effect (Figure 11(a); *P* < 0.01, Figure 11(b); *P* < 0.001).

Similarly, immunofluorescence staining results showed that the A*β*_1-42_ treated cells had an increased PARKIN fluorescence intensity (Figures 11(e) and 11(f); *P* < 0.01). Whereas pretreatment with CM and ALA-CM of the SH-SY5Y cells exposed to A*β*_1-42_ significantly decreased PARKIN fluorescence intensity (Figures 11(e) and 11(f); *P* < 0.01). Similarly, the pretreatment with insulin decreased PARKIN fluorescence intensity in comparison with DM + A*β*_1−42_ group (Figures 11(e) and 11(f); *P* < 0.001). However, the SH-SY5Y cells exposed to cotreatment of CM and ALA-CM with IDE showed an increased PARKIN fluorescence intensity in comparison to CM (Figures 11(e) and 11(f); *P* < 0.01) and ALA-CM groups (Figures 11(e) and 11(f); *P* < 0.05).

Removal of damaged mitochondria requires also the induction of general autophagy [22]. Therefore, we assessed expression levels of ATG5 and LC3*β*, well-known markers of autophagy. The RT-qPCR results revealed that A*β*_1-42_ exposure resulted in elevated mRNA levels of *ATG5* and *LC3β* (Figures 11(c) and 11(d); *P* < 0.001). Whereas pretreatment with CM of the SH-SY5Y cells exposed to A*β*_1-42_ significantly decreased expression of *ATG5* (Figure 11(c); *P* < 0.01) and *LC3β* (Figure 11(d); *P* < 0.001) in comparison with DM + A*β*_1−42_ group.

Interestingly, ALA preactivated CM significantly intensified this effect (Figure 11(c); *P* < 0.01, Figure 11(d); *P* < 0.001).

Besides, the SH-SY5Y cells exposed to the cotreatment of CM and ALA-CM with IDE showed increased expression of mitophagy and autophagy markers (Figures 11(a)–11(d); *P* < 0.001).

**Figure 12:**
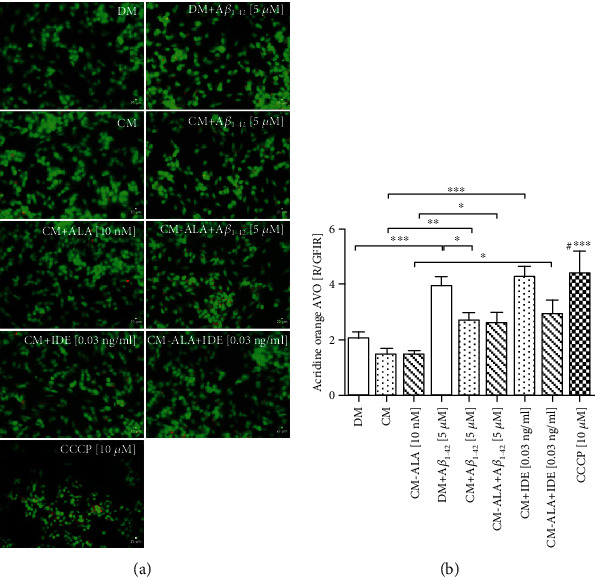
The CM and ALA-CM pretreatment reduced acidic vesicular organelles (AVOs) production in the SH-SY5Y cells treated with Amyloid *β* (A*β*_1-42_). Representative fluorescence microscopy images of acridine orange (AO) staining (a) and the ratio of red to green fluorescence intensity ratio (R/GFIR) (b) were used to measure the late state of autophagy, which is characterized by AVOs production. On the day 6th, the SH-SY5Y cells (differentiated) were pretreated for 1 h with CM or ALA-CM before the addition of 5 *μ*M A*β*_1-42_ for the next 24 h. The SH-SY5Y cells were also exposed to the cotreatment of CM and ALA-CM with Insulin Degrading Enzyme (IDE) to check whether insulin and IGF-I presence in CM and ALA-CM was responsible for the neuroprotective effect. The positive control was the SH-SY5Y cells treated with carbonyl-cyano-m-chlorophenylhydrazone-CCCP (10 *μ*M). Next, the SH-SY5Y cells were subjected to vital staining with AO (see Materials and Methods section). Red to green fluorescence intensity ratio (R/GFIR) was calculated in at least 10 replicates for each treatment and nontreated controls. One-way ANOVA followed by Tukey's multiple comparisons test at the 0.05 level was used to determine differences between the treated cells and untreated control cells. Results are presented as means ± SEM. Statistical differences between the treated cells and untreated control cells are indicated by asterisks (^∗^ for *P* < 0.05; ^∗∗^ for *P* < 0.01; ^∗∗∗^ for *P* < 0.001; ^#^^∗∗∗^ versus the control group).

The late state of autophagy is characterized by the development of acidic vesicular organelles (AVOs), which include lysosomes as well as autophagosomes. Therefore, to determine the late state of autophagy, the SH-SY5Y cells were stained with acridine orange (AO). The formation of punctate staining was monitored with fluorescence microscopy. As shown in Figures 12(a) and 12(b), A*β*_1-42_ treatment markedly increased AVO production (*P* < 0.001). Whereas pretreatment with CM and ALA-CM remarkably reduced AVO production in the SH-SY5Y cells treated with A*β*_1-42_ to similar values of untreated cells (*P* < 0.05). Increased AVO production was also observed in the SH-SY5Y cells exposed to the cotreatment of CM and ALA-CM with IDE.

## 4. Discussion

The main purpose of the present study was to explore indicated a new alpha-linolenic acid- (ALA-) induced mechanism of neuroprotection during A*β*-associated neuronal damage, with special regard to astrocyte involvement. Herein, we reported that ALA stimulated the secretory activity of astrocytes. Moreover, our results showed that ALA preactivated astrocytes conditioned medium reversed A*β*_1-42_-induced mitochondrial dysfunction and neuronal death.

*ω*-3 (n-3) polyunsaturated fatty acids (PUFAs) are fatty acids that are important for human health, especially for brain development and function. Alpha-linolenic acid, the most abundant n-3 PUFA, is an essential fatty acid in the human diet and is present in green leaves, oil, seeds (flaxseed, canola, perilla), and nuts. Various studies have suggested that ALA exerts neuroprotective and anti-inflammatory effects [11–13]. However, the direct effect of ALA on the trophic activity of astrocytes has not been studied yet.

In the current study, we focused on astrocytes because it is widely accepted now that they play a key role in the central nervous system. Astrocytes produce and release multiple proteins that impact the survival, migration, differentiation, and function of neurons. Importantly, these proteins can serve as neurotrophic agents against A*β* toxicity [7, 8, 23]. In this study, we focused our interest on insulin and IGF-I as likely candidates for neuroprotection, taking into account their involvement in synaptic and mitochondrial function, as seen also in AD. It seems that insulin and IGF-I production in the brain may be a controversial topic. For many years, it has been thought that brain insulin was derived from pancreatic *β* cells and permeated through the blood-brain barrier [24]. However, in the last few years, this hypothesis has been abolished. Nowadays, it is believed that astrocytes may be the source of local insulin and IGF-I in the brain. More recently, insulin and IGF-I secretion was also confirmed in cultured astrocytes [8, 25, 26]. Therefore it seems that the identification of astrocytes as an insulin and IGF-I source in the brain would allow us to better understand the importance of these factors in brain physiology, and it could also point to a new therapeutic target for dementia and age-related cognitive disorders. Interestingly, in the present study, we demonstrated for the first time that ALA stimulates astrocytes to express and release insulin and IGF-I to the medium (Figures 3(c) and 3(d)). However, we also observed that ALA at higher concentrations reduces the viability and thus the secretory activity of astrocytes, which could be due to the excessive oxidation of ALA which generates various metabolites as well as reactive oxygen species. Demar et al. described that radiolabelled ALA entering the brain is almost completely metabolized to its *β*-oxidation products [27]. Recently, it has been suggested that the oxidation of polyunsaturated fatty acids could be associated with induced cell death. For example, Liu et al. indicated that DHA hydroperoxide is a potential inducer of apoptosis via mitochondrial dysfunction in human neuroblastoma SH-SY5Y cells [28]. Probably, the effect of ALA on astrocytes, similarly to the impact of another PUFA, could be dose-dependent. Therefore, these results indicate that ALA in lower concentrations may regulate the trophic activity of astrocytes.

The next aim of our study was to investigate whether pretreatment with CM and ALA-preactivated CM modulates A*β*_1-42_-induced cytotoxicity in differentiated SH-SY5Y cells. A*β* is a key molecular factor in the etiology of AD [29]. Previous *in vitro* studies have shown that A*β* is toxic to neurons, which is manifested by cell death [8, 29–31]. In agreement with the previous findings, our results showed that A*β*_1-42_ was highly toxic to differentiated SH-SY5Y cells what was exemplified by the reduction of MTT values in a dose-dependent manner (Figure 4). Moreover, A*β*_1-42_ probably induced apoptosis of differentiated SH-SY5Y cells. It was evidenced by morphological and biochemical alterations associated with apoptosis including condensed chromatin (Figure 6(e)) increased LDH release and increased ratio fluorescence of PI/Hoechst (Figures 6(a) and 6(b)). However, we are aware that further research is needed to determine which apoptosis pathways are activated in A*β*_1-42_-treated differentiated SH-SY5Y cells. Nonetheless, our results showed that pretreatment with CM and ALA-CM was able to attenuate the toxic effect of A*β*_1-42_. Additionally, the protective effect of CM was intensified by pre-activation with ALA.

The presented protective action of CM and ALA-CM could also effect through the regulation of synaptic proteins and rescue of synaptic function. The pathogenesis of AD correlates with neuronal dysfunction and loss of functional synapses caused by changes in neurite morphology. *In vitro* studies indicated that mechanisms of synapse deterioration in AD result from the toxic activity of A*β* [8, 32]. In our study, the addition of A*β*_1-42_ reduced levels of synaptic markers (Figures 7(a)–7(f)) and induced the fragmentation of neuritis (Figures 7(d) and 7(f)) in differentiated SH-SY5Y cells. Whereas treatment with CM and ALA-CM was able to attenuate the synaptotoxic effect of A*β*_1-42_.

Mitochondria are organelles that may activate apoptosis when their function is damaged. A recent study demonstrated that mitochondrial dysfunction is a hallmark of A*β*-induced neuronal toxicity in AD [3, 33]. Therefore, in the next step, we investigated whether mitochondria were involved in the protective effect of CM and ALA-CM against Ai_1-42_. In our study, we demonstrated that A*β*_1-42_ disrupted the function of mitochondria. Our results, in concordance with previously reported data, showed that A*β*_1-42_ significantly reduced *Δψ*m (Figures 8(a) and 8(b)) when compared to the control group [34, 35]. However, pretreatment with CM and ALA-CM reversed the A*β*_1-42_-induced depolarization of the mitochondrial membrane in differentiated SH-SY5Y cells.

Moreover, we observed the reduced mitochondrial mass in A*β*_1-42_-treated SH-SY5Y cells (Figure 9). The reduced mitochondrial mass has already been found in brains from AD when compared to a healthy brain in a mouse model of AD as well as in AD cellular models [36, 37]. A reduction in the number of mitochondria may result from impairment of the mitochondrial biogenesis or increased mitochondrial-specific autophagy clearance known as mitophagy. Our results showed that A*β*_1-42_ suppressed mRNA levels of *PGC-1α* and *mTFA*, key regulators of mitochondrial biogenesis (Figures 10(a) and 10(b)). Whereas pretreatment with CM and ALA-CM increased *PGC-1α* and *mTFA* mRNA levels (Figures 10(a) and 10(b)), reversing the A*β*_1-42_-mediated reduction of the mitochondrial biogenesis.

Another key mitochondrial functions, the mitochondrial dynamics such as fusion and fission processes, were found to be unbalanced in AD. Several findings indicate that A*β* might play a role in impaired mitochondrial dynamics [38]. In the present study, we showed that the SH-SY5Y cells incubated with A*β*_1-42_ presented alterations in the mitochondrial dynamics towards more fission rather than fusion events. The CM and ALA-CM pretreatment restored A*β*_1-42_-reduced mRNA levels of the mitochondrial profusion genes *Mfn2* (Figure 10(c)) and *Opa1* (Figure 10(d)), and, conversely, it rescued A*β*_1-42_-increased mRNA levels of the fission gene *Drp1* (Figure 10(e)). The balance between fusion and fission processes is essential to maintain the health of the neuronal cells. Moreover, Mfn2, whose expression is induced by PGC-1*α*, regulates not only the mitochondrial fusion but also mitochondrial biogenesis and mitochondrial function through changes in the mitochondrial membrane potential and the expression of mitochondrial oxidative phosphorylation subunits [39].

As observed above, decreased levels of mRNA of mitochondrial profusion genes and increased mRNA levels of mitochondrial profission genes may result in the mitophagy of damaged mitochondria. Under physiological conditions, mitophagy plays an essential role in the basal mitochondrial turnover and maintenance. The PTEN-induced putative kinase protein 1- (PINK1-) Parkin-mediated mitophagy is the most extensively studied and the best-understood mitophagy pathway [40, 41]. It has been reported that acute depolarization of mitochondrial *Δψ*m *in vitro* with *Δψm* dissipation reagents induces Parkin-mediated mitophagy and subsequently eliminates depolarized mitochondria within the autophagy-lysosomal system. Our results showed that A*β*_1-42_ significantly increased mRNA levels of *PINK-1* (Figure 11(a)) and *PARKIN* (Figure 11(b)) in comparison to the control group. Parkin-mediated mitophagy induction has been associated with reduced levels of mitochondrial outer membrane proteins including TOMM20 [42], and this observation is consistent with our results (Figures 9(a) and 9(b)). Enhanced mitophagy has been also confirmed in AD patient brains, and it has been accompanied by depletion of cytosolic PARKIN over disease progression [43]. In the recent study, we demonstrated that CM and ALA-CM abolished A*β*_1-42_ -induced mitophagy by reduction in *PINK1* and *PARKIN* levels to similar values to the control.

Removal of damaged mitochondria requires also the induction of general autophagy [22]. A*β*_1–42_ has been also reported as an autophagy inducer accompanied by increased LC3-II expression [44]. Our results also confirmed that A*β*_1-42_ increased mRNA levels of *ATG5* (Figure 11(c)) and *LC3β* (Figure 11(d)), well-known markers of autophagy, as well as increased acidic vesicular organelles production (Figures 12(a) and 12(b)). Whereas pretreatment with CM and ALA-CM abolished A*β*_1-42_-induced autophagy by reduction of *ATG5* and *LC3β* levels to similar values to the control.

Finally, to check whether insulin and IGF-I presence in CM and ALA-CM was responsible for the above described neuroprotective effects, we treated CM and ALA-CM with the insulin-degrading enzyme, which degrades both insulin and IGF-I [8]. Our results showed that IDE attenuated neuroprotective effects of CM and ALA-CM by increasing cell death (Figures 5, 6(a), 6(b), and 6(e)), decreasing *Δψm* (Figures 8(a) and 8(b)), increasing mitochondrial dysfunctions and consequently increasing mitophagy and autophagy processes. Besides, we examined the direct neuroprotective effect of insulin on differentiated SH-SY5Y cells untreated or treated with A*β*_1-42_. Our results confirm its neuroprotective effect. Moreover, our results are consistent with previously published results which have shown that both IGF-I and insulin were involved in the pathogenesis of AD. Lower serum levels of IGF-I were associated with an increased risk of developing AD [45]. Moreover, it has been reported that physiological protection against synaptotoxic A*β* can be mediated by astrocyte-derived insulin/IGF-I [8]. Besides, insulin resistance results in mitochondrial dysfunction and excessive autophagy, and in extreme cases, mitochondrial dysfunction can even lead to neuronal cell death as previously described in cases of age-associated neurodegenerative diseases like Alzheimer's disease [46].

To conclude, the present study showed that astrocyte-derived insulin/insulin-like growth factor I suppresses A*β*_1-42_-induced cytotoxicity in the SH-SY5Y cells by protecting against mitochondrial dysfunction. Moreover, the neuroprotective effects of CM are intensified by preactivation with ALA. Our study suggests that ALA may be a very promising AD-modifying drug candidate.

## 5. Conclusions

1. Alpha-linolenic acid stimulates the release of insulin and IGF-I from astrocytes
2. Astrocyte-derived insulin/IGF-I protects differentiated SH-SY5Y cells against A*β*_1-42_- induced cell death
3. Astrocyte-derived insulin/IGF-I protect against A*β*_1-42_-induced mitochondrial dysfunction in differentiated SH-SY5Y cells via:

- reducing depolarization of the mitochondrial membrane
- increasing mitochondrial biogenesis and restoring the balance between fusion and fission processes
- regulation of mitophagy and autophagy processes

## Figures and Tables

**Table 1:** Primers and their sequences used to identify genes in the quantitative real-time reverse-transcription-polymerase chain reaction (RT-qPCR).

Gene name	Forward primer sequences	Reverse primer sequences
Mitophagy genes		
*PINK1*	GGACGCTGTTCCTCGTTA	ATCTGCGATCACCAGCCA
*PARKIN*	CCCACCTCTGACAAGGAAACA	TCGTGAACAAACTGCCGATCA
Autophagy genes		
*ATG5*	ATGATAATGGCAGATGACAAGG	TCAGTCACTCGGTGCAGG
*LC3β*	AAAGGAGGACATTTGAGCAG	AATGTCTCCTGGGAAGCGTA
Mitochondrial biogenesis genes		
*TFAM*	GTGGGAGCTTCTCACTCTGG	TAGGGCTTTTTCTCCTGCAA
*PGC1α*	CACCAGCCAACACTCAGCTA	GTGTGAGGAGGGTCATCGT
Mitochondrial dynamics genes		
*MFN2*	AATCTGAGGCGACTGGTGA	CTCCTCCTGTTCGACAGTCA
*OPA1*	GGCCAGCAAGATTAGCTACG	ACAATGTCAGGCACAATCCA
*DRP1*	GGCAACTGGAGAGGAATGC	CTTTTTGTGGACT
Synaptic genes		
*PSD95*	CTTCATCCTTGCTGGGGGTC	TTGCGGAGGTCAACACCATT
*Synaptophysin*	CTGCGTTAAAGGGGGCACTA	ACAGCCACGGTGACAAAGAA
Reference gene		
*GAPDH*	GAAGGTGAAGGTCGGAGT	GAAGATGGTGATGGGATTTC

## Data Availability

Please contact with the main author of the work by email. alitwiniuk@cmkp.edu.pl.
